# Transmissible Viral Proventriculitis Caused by Chicken Proventricular Necrosis Virus Displaying Serological Cross-Reactivity with IBDV

**DOI:** 10.3390/ani11010008

**Published:** 2020-12-23

**Authors:** Marcin Śmiałek, Michał Gesek, Daria Dziewulska, Jowita Samanta Niczyporuk, Andrzej Koncicki

**Affiliations:** 1 Department of Avian Disease, Faculty of Veterinary Medicine, University of Warmia and Mazury, ul. Oczapowskiego 13/14, 10-719 Olsztyn, Poland; daria.pestka@gmail.com (D.D.); koncicki@uwm.edu.pl (A.K.); 2 Department of Pathological Anatomy, Faculty of Veterinary Medicine, University of Warmia and Mazury, ul. Oczapowskiego 13, 10-719 Olsztyn, Poland; michal.gesek@uwm.edu.pl; 3 Department of Poultry Diseases, National Veterinary Research Institute in Puławy, 57 Partyzantów Avenue, 24-100 Puławy, Poland; jowita.niczyporuk@piwet.pulawy.pl

**Keywords:** transmissible viral proventriculitis, chicken proventricular necrosis virus, infectious bursal disease virus, fowl adenovirus

## Abstract

**Simple Summary:**

In our study we were trying to evaluate the etiological agent behind transmissible viral proventriculitis in broiler chickens—the disease that causes severe losses to the poultry industry. The results of our study confirm the hypothesis that the disease is caused by chicken proventricular necrosis virus (CPNV), a member of the Birnaviridae family. Additionally, we have discovered that after CPNV infection, birds produce antibodies that can be detected with the use of a commercial test, which is specific against other, well-characterized Birnaviridae family member. The results of our study shed a new light on the subjects concerning the diagnosis and prevention of transmissible viral porventriculitis in chickens.

**Abstract:**

Transmissible viral proventriculitis (TVP) of chickens is manifested in decreased body weight gains, poor feed conversion and weight diversity. Although TVP etiology has not been defined, a *Birnaviridae* family member, named chicken proventricular necrosis virus (CPNV) is considered as a potential factor of a disease. This study was undertaken in order to reproduce TVP and to evaluate its etiology. Broiler chickens of the TVP-infected group were inoculated with TVP positive proventriculi homogenate on the 24th day of life. Samples were collected, on infection day and 14 days post-infection (dpi). The 14 dpi anatomo- and histopathological evaluation, revealed that we have succeeded to reproduce TVP. TVP-infected birds gained 30.38% less body weight. In the TVP-infected group a seroconversion against picornaviruses, fowl adenoviruses (FAdV) and infectious bursal disease viruses (IBDV) was recorded with an ELISA test. Using RT-PCR and PCR, CPNV was detected in proventriculi and FAdV in spleens and livers of infected birds, 14 dpi. Our study supports that CPNV is involved in the development of TVP. We did not record the presence of IBDV in TVP or control birds, despite our recording of a seroconversion against IBDV in TVP infected birds. CPNV and IBDV belong to the same family, which allows us to assume serological cross-reactivity between them. The role of FAdV needs further evaluation.

## 1. Introduction

Transmissible viral proventriculitis (TVP) is an infectious disease reported in the production of all types of chickens and has significant impact on the poultry industry. The typical pathological lesions observed in the course of TVP affect the proventriculus and are described as proventricular enlargement, thickening of its walls and spotty discoloration in the cross-section. In isolated cases, small hemorrhagic changes are observed in the proventricular mucosa [1].

The first reports on TVP date back to 1978 and come from the Netherlands [2], when Kouwenhoven et al. reported a case of proventriculitis in commercial chicken broilers and proved that TVP was induced by an infectious factor. Since then, TVP cases have been identified and reported in the USA, Australia, China, South Korea, Spain, France, the UK, and Poland, among other countries [3,4,5,6,7,8,9,10].

The etiology of TVP has not been explicitly defined so far. Studies on TVP etiopathology imply the involvement of infectious bursal disease viruses (IBDV) of the *Birnaviridae* family, IBDV-like viruses, infectious bronchitis viruses (IB) of the *Coronaviridae* family, reoviruses (REO), picornaviruses, fowl adenoviruses (FAdV), adeno-like viruses, or mixed infections in the development of typical pathological lesions [3,4,5,6,8,10,11,12,13,14,15]. Recently, a novel virus identified as a member of the *Birnaviridae* family has been isolated from clinical cases of TVP. Preliminary studies confirmed that this virus differed significantly from the *Avibirnavirus* genus IBDV. This virus was named chicken proventricular necrosis virus (CPNV), and an RT-PCR method has been developed which enables its detection [13]. Unfortunately, cases of TVP which were negative for CPNV as well as cases positive for CPNV presence without typical TVP changes have also been reported [12], which makes the etiology of TVP unresolved and further research is still necessary.

Clinically, TVP is manifested mainly in broiler chickens by decreased body weight gains, wide weight diversity of birds in the flock and an increased feed conversion ratio [1]. The disease, which usually affects up to 50% of the birds in the flock, can significantly reduce the cost-effectiveness of production.

Considering its problematic etiology, TVP diagnosis is difficult. Histopathological examination seems to be the most reliable method for confirming the disease. The histopathological lesions observed in the case of TVP exclusively affect the proventriculus and are manifested in a triad of lesions related to the necrosis of glandular epithelial cells (even up to 80% of cells in the proventricular mucosa), a strong lymphatic infiltration in the lamina propria of the mucosa and among the proventricular glands, hypertrophy of the epithelial cells of the excretory ducts of proventricular glands with successive replacement of the epithelial glandular cells with hypertrophied cells of the excretory ducts. The severity of these lesions can vary depending on the duration of the disease [1,12,16].

Given the recent cases of TVP recorded in Poland [9], a laboratory-conditions study was undertaken that attempted to reproduce the clinical course of TVP in broiler chickens by inoculating them with a homogenate of proventriculi from a confirmed TVP field case. The research was also aimed at identifying the etiological factor in domestic cases of TVP.

## 2. Materials and Methods

### 2.1. Ethic Statement

The experimental procedures and animal handling procedures were conducted with the approval of the Local Ethic Committee for Animal Experiments in Olsztyn, Poland (resolution No. 50/2017). The study was carried out in accordance with EU Directive 2010/63/EU on the protection of animals used for scientific purposes.

### 2.2. Birds

Commercial Ross 308 broiler chicks of both sexes, purchased from one hatchery and one hatch, were used in the experiment. The trial was conducted in isolated pens of the Pavilion of Experimental Poultry Infections, at the Department of Avian Diseases, University of Warmia and Mazury in Olsztyn, which are maintained at a biosafety PCL 3 (Physical Containment Laboratory 3) conditions. Water and feed were given to birds ad libitum. Rearing conditions were consistent with Aviagen (Aviagen, NY, USA) recommendations.

### 2.3. Experiment Layout

The experiment was carried out with 24 commercial broiler chickens. The birds were reared until day 24 of life, after which they were divided into two groups (*n* = 12). One served as the control, and the other was infected with a proventriculi homogenate originating from a confirmed field TVP case. Before the infection (0 days post infection—dpi), 4 birds selected randomly from each group were euthanized and subjected to autopsy examination to check for macroscopic lesions typical of TVP. Afterwards, proventriculi were collected for histopathological examination, and samples of them and of other internal organs (intestines, spleen, liver, and bursa of Fabricius) were taken for molecular analysis. For histopathological examination, the proventriculi samples were fixed in a 10% formaldehyde solution. Samples for molecular analysis were frozen at −20 °C until they were analyzed. The remaining birds from both groups were weighed and blood samples were collected for serological analysis. Blood was centrifuged at 1500× *g* for 15 min and the serum obtained was frozen at −20 °C until it was analyzed. After infection, the birds were reared for the next 14 days. At 14 dpi, blood was sampled from all birds from both groups for serological analysis. Next, the birds were weighed and euthanized. Pathological lesions in the proventriculi were investigated and recorded during the anatomopathological examination, after which samples of this organ were collected for histopathological and molecular examinations, together with other internal organs, as described for 0 dpi. Production results were presented as the mean body weight (kg) +/− standard deviation (SD).

For euthanasia, chickens were placed in a chamber with Carbogen (95% O_2_ + 5% CO_2_). After 1 min, Carbogen was slowly replaced by 100% CO_2_. This method of euthanasia is responsible for stress reduction in the birds.

### 2.4. Proventriculi Homogenate and Infection

Proventriculi from the first described Polish case of TVP [9] were used for the experimental infection. The proventriculi that were used for the experimental infection in our study were collected from 5-week-old Ross 308 broiler chickens from a commercial farm and they were confirmed by histopathological examination to be TVP-positive. These proventriculi were stored frozen (−20 °C) prior to infection. After homogenization of defrosted samples, proventriculi were processed through three freeze–thaw cycles. After centrifugation (2000× *g*, 15 min), the supernatant was stored and used for the infection. Broiler chickens from the TVP-infected group were inoculated with 5 mL of the supernatant per bird. The supernatant was given to birds *per os*, directly with a probe to the crop. At the same time, the birds from the control group were given PBS in the same way. Additionally, samples of proventriculi, that were used for bird infection, were processed for FAdV and IBDV identification.

### 2.5. Serological analysis

Commercial kits of ELISA antibody tests (IDEXX Laboratories, Lenexa, KS, USA) were used to determine the titer of anti-IBV, anti-REO, anti-FAdV, and anti-IBDV specific IgY in broiler serum. Particular stages of the tests were performed with an Eppendorf epMotion 5075 LH automatic pipetting station (Eppendorf, Hamburg, Germany), a BioTek ELx405 automatic multi-well plate washer (BioTek, Winooski, VT, USA), and a BioTek ELx800 plate reader. Sample to positive (S/P) ratio, plus/minus standard deviation (SD) were computed for each group in each sampling period.

### 2.6. Gross Lesion Evaluation

The birds were investigated for the presence of lesions typical of TVP in the proventriculi during an anatomopathological examination. Enlargement of the proventriculi coupled with thickening of its walls and their spotty discoloration in the cross-section were recorded. Results were collated as the number of birds with recorded pathological lesions relative to the total number of birds examined.

### 2.7. Histopathology

During necropsy, samples of the central part of the proventricular wall were embedded in 10% formalin (pH 7.4) and processed for histopathological examination. After passing the samples through intermediate liquids (increasing concentrations of alcohol and xylene) they were embedded in paraffin blocks. Sections of the examined samples that were 4 μm thick were stained with hematoxylin–eosin and microscope samples were scanned with a Pannoramic MIDI scanner (3DHISTECH, Budapest, Hungary). Samples were considered TVP positive if the following histopathological lesions were registered during examination—necrosis of glandular epithelium (necrosis), hypertrophy and hyperplasia of ductal epithelium, replacement of glandular epithelium by hyperplastic ductal epithelium (hyperplasia) and multifocal-to-severe infiltration of lymphoid cells (infiltration). Three sections (one transverse through the middle of the proventriculi and two longitudinal from the middle of the proventriculi to the esophagus and to the gizzard respectively) were evaluated per every proventriculus sample. For PCR analysis, five 4 μm sections from each paraffin block (each bird) were put into xylene and prepared for PCR analysis.

### 2.8. CPNV, IBDV and FAdV Identification

Paraffin fixed sections of the proventriculi were used for CPNV identification. One mL of xylene was added to each sample comprising five paraffin-fixed proventriculi sections (each 5 µm thick) and these were incubated for 5 min at 50 °C in order to remove paraffin residues. Further RNA isolation steps were performed with an Isolate II FFPE RNA/DNA Kit (Bioline, London, UK) according to the manufacturer’s recommendations. Concentration and the purity of isolated RNA were established with the use of a NanoDrop 2000 spectrophotometer (Thermo Fisher Scientific, Waltham, MA, USA). A High-Capacity cDNA Reverse Transcription Kit (Life Technologies, Carlsbad, CA, USA) was used to transcribe the RNA. The reaction was performed with 2 µL of 10X RT Buffer, 0.8 µL of 25X dNTP Mix (100 mM), 0.5 µL of 100 µM 5′- GGGCGGTAACCATTCAGATA- 3′ reverse primer, 1 µL of MultiScribe Reverse Transcriptase, 1 µL of RNase Inhibitor, 4.7 µL of nuclease-free water and 10 µL of RNA (previously incubated for 5 min at 99 °C). The PCR was performed as described previously [13]. The amplification of the target 171 bp CPNV gene was performed with the use of a HotStarTaq Plus Master Mix Kit (Qiagen, Hilden, Germany) and carried out with 10 µL of HotStarTaq Plus DNA Polymerase, 0.1 µL of each 100 µM primer, 2 µL of CoralLoad PCR Buffer, 5.8 µL of RNase-free water and 2 µL of cDNA. After pre-denaturation at 95 °C for 5 min, the denaturation step was performed at 94 °C for 1 min, followed by primer annealing at 55 °C for 1 min, product elongation at 72 °C for 1 min, and final elongation at 72 °C for 10 min. Thirty-five replication cycles were performed.

Total RNA for IBDV identification was extracted directly from the homogenized internal organs of the examined birds. Briefly, 700 µL of sterile PBS was added to each sample (0.2 g) and then samples were homogenized with the use of a TissueLyser II (Qiagen). Samples were centrifuged at 1500× *g* for 30 s. After centrifugation, 200 µL of the supernatant was used for further steps of RNA isolation, which were performed with an RNeasy Mini Kit (Qiagen) according to the manufacturer’s recommendations. Reverse transcription was performed with the use of High-Capacity cDNA Reverse Transcription Kit (Life Technologies). IBDV was detected in the samples adopting the method described previously by Lin et al. [17] again with the use of a HotStarTaq Plus Master Mix Kit (Qiagen) and the following set of primers: IBDV F (sense primer): 5′ CCCAGAGTCTACACCATA 3′ and IBDV R (antisense primer): 5′ TCCTGTTGCCACTCTTTC 3′. The reaction was performed with 10 µL of HotStarTaq Plus DNA Polymerase, 0.1 µL of each 100 µM primer, 2 µL of CoralLoad PCR Buffer, 5.8 µL of RNase free water and 2 µL of cDNA. After pre-denaturation at 95 °C for 5 min, the denaturation step was performed at 94 °C for 1 min, followed by primer annealing at 55 °C for 1 min, product elongation at 72 °C for 1 min, and final elongation at 72 °C for 10 min. Thirty-five replication cycles were performed.

Total DNA for FAdV identification was extracted directly from the internal organs of the examined birds. Extraction was performed using a DNA Mini Kit (Qiagen) according to the manufacturer’s procedure. DNA templates were stored at −20 °C until further analysis. DNA obtained from the reference strain type/species 2/D served as a positive control. The specific oligonucleotide primers were used for the amplification of the loop L1 region of the hexon gene of all FAdV types. The primers were synthesized at the Genomed company (Warsaw, Poland). The sequences of nucleotide primers were as follows: FAdV F (forward primer)—5′ATGGGAGCSACCTAYTTCGACAT 3′ and FAdV R (reverse primer)—5′AAATTGTCCCKR AANCCGATGTA 3′. The expected product size was 830 bp. The reaction was conducted in a final volume of 25 μL, which contained 2.5 μL of 10X PCR buffer, 1 μL of dNTP (10 mM), 1.5 μL of each primer (10 μM), 2 μL of DNA template, and 11.5 μL of sterile water. After the pre-denaturation at 95 °C for 5 min, the denaturation step was performed at 94 °C for 45 s, followed by primer annealing at 55 °C for 1 min, product elongation at 72 °C for 2 min, and final elongation at 72 °C for 10 min. Thirty-five replication cycles were performed. Amplification was conducted in a basic gradient thermocycler (Biometra, Göttingen, Germany). After amplification, electrophoresis was carried out in 2% agarose gel with 1 μg/mL of ethidium bromide. Electrophoresis was conducted in Tris borate EDTA buffer, pH 8.2, (150 V and 80 mA) for 50 min in a Mini-Sub Cell (Biorad, Hercules, CA, USA). After gel electrophoresis, the size of the amplicons was compared with the MassRuler Low Range DNA ladder to 1031 bp (Fermentas/Thermo Fisher Scientific Baltics, Vilnius, Lithuania). The results were visualized using a UV transilluminator, then photographed and analyzed. The results were considered positive when the DNA product obtained had the predicted size of 830 bp.

### 2.9. Statistics

Statistical analysis was performed with GraphPad Prism 6.05 with the use of Mann Whitney *U* test. Differences were considered statistically significant at *p* < 0.05.

## 3. Results

### 3.1. Body Weight

The mean body weight of the control and TVP-infected birds is summarized in Table 1. Over a 14-day span after the infection, the birds from the control group gained 1.58 kg on average, while the birds from the TVP group gained 1.1 kg. The body weight gain of the chickens in the TVP group, after the infection, was significantly reduced by 30.38% than in the control group (*p* = 0.034).

### 3.2. Gross Lesions

**Figure 1:**
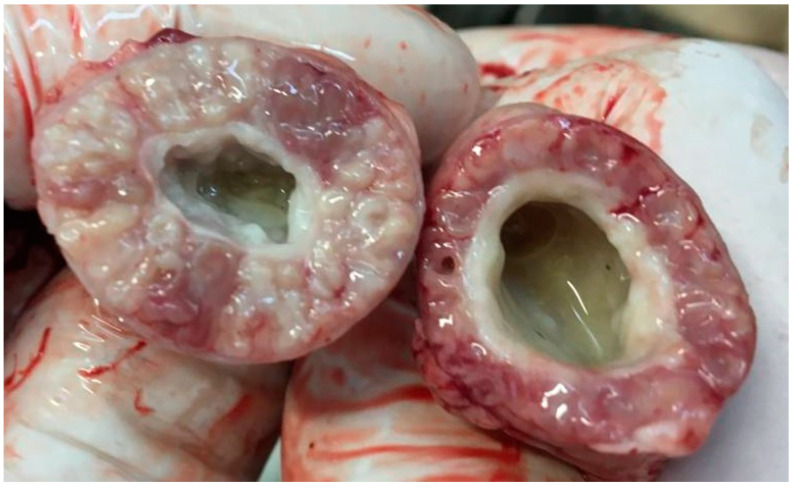
Gross lesions. Proventriculi of TVP (left) and Control (right) broiler chickens examined at 14 dpi. Typical pathological lesions observed in proventriculi of TVP infected birds were characterized as: enlargement, thickening of its walls and spotty discoloration in the cross-section, which are the three characteristic gross lesions related to transmissible viral proventriculitis. Those lesions were recorded in 5 (discoloration) or 6 (enlargement and thickening) out of 8 TVP infected birds.

No lesions typical for TVP were recorded in any of the groups at the time of infection on the 24th day of life. In the TVP group at 14 days after the infection, enlargement of the proventriculi was noted in six out of the eight birds examined, thickening of the proventricular wall was also observed in six out of eight, and discoloration was registered in five (Figure 1). No TVP lesions were recorded in the control group at this time. The relevant data are summarized in Table 2. No other anatomopathological lesions were observed in any of the birds from either the control or TVP groups.

### 3.3. Histopathological Lesions

**Figure 2:**
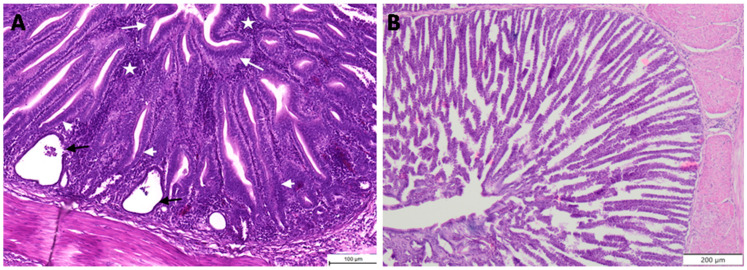
Histopathological lesions. Left—histopathological lesions typical for transmissible viral proventriculitis were recorded in 7 out of 8 examined proventriculi of birds from TVP group. The lesions concerned necrosis of glandular epithelium (black arrows), hypertrophy and hyperplasia of ductal epithelium (white arrow), replacement of glandular epithelium by hyperplastic ductal epithelium (white arrowhead) and infiltration of lymphoid cells (star) (**A**). Right—histological image of a control bird (**B**).

Histopathology revealed no TVP lesions in the proventriculi of birds from either group on the day of infection. In the TVP group, histopathological lesions associated with TVP, which were characterized as necrosis of glandular epithelium (necrosis), hypertrophy and hyperplasia of ductal epithelium, replacement of glandular epithelium by hyperplastic ductal epithelium (hyperplasia) and multifocal to severe infiltration of lymphoid cells (infiltration) were recorded at 14 dpi, in seven out of eight proventriculi examined (Figure 2). No histopathological changes were observed in the control birds. The results of the histopathological examination are provided in Table 3.

### 3.4. Serological Results

The results of the serological examination are summarized in Table 4. In the TVP group, a significant increase in the anti-FAdV and anti-IBDV antibodies level was recorded as 14 dpi in comparison to their level on the day of infection. At the same time, levels of these antibodies were significantly higher in the TVP group than in the Control group, at 14 dpi.

### 3.5. Molecular Biology

Samples of proventriculi homogenate used for bird inoculation were negative for the presence of FAdV and IBDV. The results of molecular studies performed on the samples from experimental birds are shown in Table 5. All samples collected at 0 and 14 dpi in the control group were negative in all assays. In the TVP group at 0 dpi, all samples were also negative. In this group at 14 dpi, the samples of proventriculi (100% of samples tested) were positive for CPNV, while those of spleens and livers were positive for FAdV. 

## 4. Discussion

Clinically, TVP is manifested by lower body weight gains, wide weight diversity of birds in the flock and an increased feed conversion ratio [1]. From just a few reports, describing TVP cases, we have concluded that the disease occurs in birds that are older than 3 weeks, which was the reason for the time of the inoculation in our study (24th day of the bird’s life). Additionally, the incubation period of the disease (which was not clearly defined) was estimated to last between 10 to 14 days [1,2] and that is why we have decided to collect samples after the maximum estimated incubation period. From our observations, it seems that incubation period of TVP can be even shorter as we have recorded (visually) decreased body weight gains in TVP-infected birds from day 7 after the inoculation (data not shown). Our observations are in agreement with the reports that TVP-associated histopathological lesions in chicken proventriculi can be detected 5 days after the CPNV infection [7]. Goodwin et al. [4] reported that suboptimal body weight gains could result from pepsinogen- and hydrochloric acid-producing cell destruction and that, in severe cases of TVP, 80% of these cells are destroyed due to necrosis caused by infection. In the course of TVP, no increased mortality is observed in the flock, but the number of culled birds increases significantly. In our study, no increase in mortality was manifest but poorer body weight gain was recorded in the TVP group. Additionally, in this group both the anatomopathological and histopathological changes typical of TVP were observed. Given the fact that such lesions were not recorded in the control group and that no other lesions apart from those related to TVP were observed in any of the groups examined, we may conclude that we have succeeded in the reproduction of TVP under experimental conditions. Our study showed that over the 14-day period post-infection, the body weight gains of TVP-infected birds were lower by over 30% than those of the control birds.

To date, the etiological agent causing TVP has not been clearly established. However, the infectious nature of the disease has been emphasized many times, which is reflected in our work. Studies on TVP etiopathology suggest infectious bursal disease viruses, infectious bronchitis viruses, reoviruses, picornaviruses, adenoviruses, adeno-like viruses, IBDV-like viruses, or mixed infections in the development of characteristic lesions [3,4,5,6,8,10,11,12,13,14,15]. Recently, CPNV was suggested as a possible TVP causative agent [6]. With what is currently known about CPNV, it seems that the first description of this virus comes from 1996 when Goodwin et al. [4] observed hexagonal virus particles in the nuclei and cytoplasm of proventricular cells in a case of TVP [4]. However, a more detailed description along with the taxonomy of CPNV was provided later by Guy et al. [13]. Chicken proventricular necrosis virus is a twenty-walled, non-enveloped virus with a diameter of approximately 75 nm. As genetic material, it has double-stranded RNA, organized into two segments (c. 3.8 and 3.4 kbp). Within the putative viral protein-1–encoding gene (VP1), CPNV has a motif characteristic of *Birnaviridae* encoding the RNA-dependent RNA polymerase. However, based on the phylogenetic studies of the VP1 gene, it has been established that this virus is different from other known members of this family of viruses. It is interesting that clinical TVP was reproduced with the use of isolated CPNV, and the virus was detected in proventriculi samples for up to 14 days after the inoculation [7].

Recent research from the UK also suggests the involvement of CPNV in the development of TVP in chickens. In two studies, the authors reported that CPNV was detected in 22% and 47% of confirmed clinical cases of TVP [11,12]. It remains unknown, however, if all cases of this disease are of the same infectious origin. There are cases of TVP in which CPNV cannot be identified, as well as disease cases in which CPNV can be identified but which do not meet the diagnostic criteria of TVP [7,11,12]. In addition, it is suggested that the pathogenesis of TVP could be more complex and may result from other viral, bacterial or fungal co-infections [1].

Due to seroconversion against FAdV and IBDV being observed in the studied TVP group, we performed a further molecular analysis to confirm the presence of these viruses in the samples from experimental birds and in the proventriculi used for bird infection. Additionally, we have evaluated the presence of CPNV in the proventriculi samples from experimental birds. At 14 dpi, we confirmed the presence of CPNV in 100% of the proventriculi samples collected from the TVP group, which indicates the involvement of this virus in TVP development and it is in the agreement with previous reports [7]. In addition, a retrospective study performed with formalin-fixed, paraffin-embedded proventriculi samples originating from the same field case, and collected simultaneously with proventriculi that eventually were used for bird infection in this experiment, gave positive results for CPNV presence (data not shown). In our experiment, we also confirmed the presence of FAdV in the TVP-infected birds. It is difficult to clearly state the nature of FAdV’s but it seems that CPNV infection contributed to the activation of a latent FAdV infection in the experimental birds, as FAdV was not present in the proventriculi used for bird infection. This hypothesis if further supported by the fact that FAdV was not detected in the proventriculi of TVP-infected birds but only in spleen and liver samples. In our experiment we have used commercial birds from a commercial hatchery and FAdV are known for their vertical transmission. The explanation of these relationships requires further research. Once again, these results highlight the complex nature of TVP etiology.

## 5. Conclusions

In this experiment we were able to reproduce the clinical TVP in broiler chickens under laboratory conditions with the use of homogenates of proventriculi from confirmed cases, first described in a Polish case of this disease. We have demonstrated that CPNV was involved in the development of the disease. To our surprise, we did not record the presence of IBDV in the TVP or control birds, nor in the proventriculi samples used for bird infection, despite our recording a strong seroconversion against IBDV in the birds from the TVP group. TVP etiological agent—CPNV belongs to the same *Birnaviridae* family as IBDV, which allows us to assume serological cross-reactivity between them. This should be taken into account during serological evaluation under field conditions, as the CPNV infection can influence the IBDV antibody levels detected by commonly available ELISAs. On the other hand, the possibility of using IBDV ELISA kits for TVP diagnosis might be considered. Undoubtedly, these issues, as well as the contribution of FAdV in the development of TVP, require further research.

## Figures and Tables

**Table 1:** Mean body weight of birds in transmissible viral proventriculitis (TVP) and Control groups.

Group	Mean Body Weight (kg) ± SD at Different Time Points after Infection	
	0 dpi	14 dpi
Control	1.54 ± 0.14	3.12 ± 0.23
TVP	1.56 ± 0.16	2.66 ± 0.42 *

* significant difference in the mean body weight between birds of the TVP and the control group at the same dpi.

**Table 2:** Prevalence of gross lesion in birds of TVP and Control groups.

Gross Lesion Recorded in Proventriculi	Number of Birds in the Group with Lesions at Different dpi			
	Control		TVP	
	0 dpi	14 dpi	0 dpi	14 dpi
Enlargement	0/4	0/8	0/4	6/8 */**
Thickening	0/4	0/8	0/4	6/8 */**
Discoloration	0/4	0/8	0/4	5/8 */**

* significant difference in the number of proventriculi with gross lesion in TVP group in comparison to the Control group at the same dpi. ** significant difference in the number of proventriculi with gross lesion within the group (TVP or Control) at 14 dpi in comparison to 0 dpi.

**Table 3:** Prevalence of histopathological lesion in birds of TVP and Control groups.

Histopathological Lesion Recorded in Proventriculi	Number of Birds in the Group with Lesions			
	Control		TVP	
	0 dpi	14 dpi	0 dpi	14 dpi
Necrosis	0/4	0/8	0/4	7/8 */**
Hyperplasia	0/4	0/8	0/4	7/8 */**
Infiltration	0/4	0/8	0/4	7/8 */**

* significant difference in the number of proventriculi with histopathological lesion in TVP group in comparison to the Control group at the same dpi. ** significant difference in the number of proventriculi with histopathological lesion within the group (TVP or Control) at 14 dpi in comparison to 0 dpi.

**Table 4:** Mean IB, reoviruses (REO), picornaviruses, fowl adenoviruses (FAdV) and infectious bursal disease viruses (IBDV) antibody levels ± SD in Control and TVP group at different dpi. In birds of the TVP-infected group, a strong seroconversion against IBDV and FAdV was recorded at 14 dpi.

Group	Parameter	Mean S/P ± SD (Positive/Tested) at Different Time Points after Infection							
		IB		REO		FAdV		IBDV	
		0 dpi	14 dpi	0 dpi	14 dpi	0 dpi	14 dpi	0 dpi	14 dpi
**Control**	S/P	0.08	0.188	0.624	0.404	0.126	0.118	0.093	0.036
	SD	0.049	0.098	0.013	0.026	0.053	0.056	0.045	0.060
	pos/tested	0/8	0/8	0/8	0/8	0/8	0/8	0/8	0/8
**TVP**	S/P	0.060	0.212	0.760	0.399	0.092	0.536 */**	0.044	0.856 */**
	SD	0.121	0.177	0.051	0.055	0.043	0.327	0.043	0.881
	pos/tested	0/8	0/8	0/8	0/8	0/8	4/8	0/8	8/8

* significant difference in the specific antibody level in TVP group in comparison to the Control group at the same dpi. ** significant difference in the specific antibody level within the group (TVP or Control) at 14 dpi in comparison to 0 dpi.

**Table 5:** Results of molecular studies. Table presents the results of IBDV, FAdV and chicken proventricular necrosis virus (CPNV) prevalence in the internal organs of birds of Control and TVP groups at different dpi.

Group	Internal Organ Tested	Number of Positive Samples/Tested at Different dpi					
		IBDV		FAdV		CPNV	
		0 dpi	14 dpi	0 dpi	14 dpi	0 dpi	14 dpi
Control	Proventriculus	0/4	0/4	nd. *	0/4	0/4	0/4
	Liver	0/4	0/4	nd.	0/4	nd.	nd.
	Spleen	0/4	0/4	nd.	0/4	nd.	nd.
	Intestines	0/4	0/4	nd.	0/4	nd.	nd.
	BF	0/4	0/4	nd.	nd	nd.	nd.
TVP	Proventriculus	0/4	0/4	nd.	0/4	0/4	4/4
	Liver	0/4	0/4	nd.	3/4	nd.	nd.
	Spleen	0/4	0/4	nd.	3/4	nd.	nd.
	Intestines	0/4	0/4	nd.	0/4	nd.	nd.
	BF	0/4	0/4	nd.	nd.	nd.	nd.

* Not done (nd.)

## Data Availability

The data presented in this study are available on request from the corresponding author.
